# Race, Poverty Status at Birth, and DNA Methylation of Youth at Age 15

**DOI:** 10.31586/gjeid.2024.988

**Published:** 2024-07-12

**Authors:** Shervin Assari, Hossein Zare

**Affiliations:** 1 Department of Internal Medicine, Charles R. Drew University of Medicine and Science, Los Angeles, CA, United States; 2 Department of Family Medicine, Charles R. Drew University of Medicine and Science, Los Angeles, CA, United States; 3 Department of Urban Public Health, Charles R. Drew University of Medicine and Science, Los Angeles, CA, United States; 4 Marginalization-Related Diminished Returns (MDRs) Center, Los Angeles, CA, United States; 5 Department of Health Policy and Management, Johns Hopkins Bloomberg School of Public Health, Baltimore, MD, United States; 6 School of Business, University of Maryland Global Campus (UMGC), College Park, United States

**Keywords:** Epigenetic aging, DNA methylation, socioeconomic status, race, Fragile Families, Child Wellbeing Study, GrimAge, health disparities

## Abstract

Epigenetic studies, which can reflect biological aging, have shown that measuring DNA methylation (DNAm) levels provides new insights into the biological effects of social environment and socioeconomic position (SEP). This study explores how race, family structure, and SEP (income to poverty ratio) at birth influence youth epigenetic aging at age 15. Data were obtained from the Future of Families and Child Wellbeing Study (FFCWS) cohort, with GrimAge used as a measure of DNAm levels and epigenetic aging. Our analysis included 854 racially and ethnically diverse participants followed from birth to age 15. Structural equation modeling (SEM) examined the relationships among race, SEP at birth, and epigenetic aging at age 15, controlling for sex, ethnicity, and family structure at birth. Findings indicate that race was associated with lower SEP at birth and faster epigenetic aging. Specifically, income to poverty ratio at birth partially mediated the effects of race on accelerated aging by age 15. The effect of income to poverty ratio at birth on DNAm was observed in male but not female youth at age 15. Thus, SEP partially mediated the effect of race on epigenetic aging in male but not female youth. These results suggest that income to poverty ratio at birth partially mediates the effects of race on biological aging into adolescence. These findings highlight the long-term biological impact of early-life poverty in explaining racial disparities in epigenetic aging and underscore the importance of addressing economic inequalities to mitigate these disparities. Policymakers should focus on poverty prevention in Black communities to prevent accelerated biological aging and associated health risks later in life. Interventions aimed at eliminating poverty and addressing racial inequities could have significant long-term benefits for public health. Future research should explore additional factors contributing to epigenetic aging and investigate potential interventions to slow down the aging process. Further studies are needed to understand the mechanisms underlying these associations and to identify effective strategies for mitigating the impact of SEP and racial disparities on biological aging.

## 1. Introduction

Epigenetic clocks measure epigenetic aging based on DNA methylation (DNAm) levels at multiple cytosine-phosphate-guanine dinucleotides (CpGs) across the genome [1]. These tools are commonly utilized to document biomarkers of aging, which is also under influence socioeconomic position (SEP) [2]. Epigenetic age acceleration (AA) indicates an older epigenetic age relative to chronological age is predictive of several age-related diseases [1].

Epigenetic age can be estimated from various tissues such as blood or saliva [3]. Over the past decade, several advances have been made available epigenetic clock algorithms used in research [4]. The first-generation epigenetic clocks, including HorvathAge and HannumAge, predict chronological age using specific CpG sites strongly associated with chronological age [5]. Second-generation clocks, such as PhenoAge and GrimAge, incorporate additional age-related clinical markers [6,7]. The residual from regressing epigenetic age on chronological age is referred to as epigenetic age acceleration (AA) [8]. More recently, a third generation, the Pace of Aging (DunedinPACE), was developed [9]. Unlike prior generations, DunedinPACE incorporates longitudinal within-person physiological changes along with methylation patterns, specifically designed to measure the rate of aging. This measure uses 173 CpG sites in blood or saliva samples [9].

Second-generation clocks outperform first-generation clocks in predicting the risk of age-related diseases and all-cause mortality, and third-generation clocks outperform second-generation clocks [10,11]. However, it is unknown which generation is more useful to detect the effects of SEP and particularly poverty on biological aging. Using the third-generation clock, DunedinPACE, a recent study documented the positive effects of high levels of education on the pace of aging, showing benefits irrespective of individuals’ genetics ^12^. The study used data from five studies across the lifespan and showed that high educational attainment is associated with a slower pace of aging even after accounting for genetic factors (meta-analysis effect size = −0.20; p = .006). This protective effect also persisted after controlling for tobacco smoking (meta-analysis effect size = −0.13; p = .01) [12].

This study explores the associations between race, SEP (income to poverty ratio) at birth and epigenetic aging at age 15. To be more specific, we tested whether SEP (income to poverty ratio) at birth partially mediates racial disparities in epigenetic aging at age 15. We used a DNAm measure namely GrimAge in the context of the Future of Families and Child Wellbeing Study (FFCWS) that is a birth cohort with data at birth and at 15 years of age.

## 2. Materials and Methods

### 2.1. Design and Setting

The Future of Families and Child Wellbeing Study (FFCWS), formerly known as the Fragile Families and Child Wellbeing Study, is a pioneering research project aimed at understanding the challenges faced by economically disadvantaged families in the United States. The FFCWS follows a birth cohort starting in 1998, tracking children from birth to youth at age 15 and then young adulthood at age 22. Detailed information on the study’s sampling techniques and methodologies can be found in previously published literature. This section provides a brief overview of the FFCWS research design.

### 2.2. Ethics

The study protocol was approved by the Institutional Review Board at Princeton University. Informed consent was obtained from all participating families, with parents or legal guardians consenting on behalf of minors, who also provided their assent. All data collection, storage, and analysis procedures were designed to protect participants’ anonymity, and families were compensated for their participation.

### 2.3. Sample and Sampling

The FFCWS recruited a diverse sample of urban families from 20 major U.S. cities, each with a population exceeding 200,000. The study specifically targeted underrepresented families, particularly non-married, Black, and Hispanic families. Consequently, the study’s sample predominantly consists of low socioeconomic status families, with a substantial representation of Black and Hispanic participants, which does not reflect the overall U.S. population. The analytical sample included 854 families that included parents and offsprings who were followed from birth to age 15 of the child. We included participants regardless of race/ethnicity, however, only those with DNAm data at age 15 were included in our analysis [13].

### 2.4. Process

Our analysis utilized data from the first and seventh waves of the FFCWS. Socioeconomic position (SEP) data were collected at birth (wave 1, baseline), and outcomes were measured when the offspring were youth at age 15 (wave 6). The analysis included 854 Black and White families with follow-up epigenetic data at age 15.

### 2.5. Predictors

Baseline data were collected through interviews with both parents, covering parents’ poverty status and family structure at birth. Family poverty status (income to poverty ratio) at birth was measured as a continuous measure ranging from 0 to 12.3, with higher number indicator of higher SEP.

### 2.6. Collection of Saliva Sample

During the Year 15 follow-up wave, saliva was collected from the focal children (now teenagers) using Oragene DNA Self-Collection Kits (OGR-500) as described for the year 9 followup with the following modifications. For those who did not complete a home visit, saliva collection kits were sent to participants via mail and after collection participants returned the kits to Westat via FedEx. Participants were discouraged from eating or drinking anything within 30 minutes prior to sample collection. Upon completion of the saliva collection, all participants received $20 [13].

### 2.7. Acquisition and Processing of the DNA Methylation Data

For the DNA methylation analysis, approximately 500 ng of genomic DNA, quantified using the Quant-iT Picogreen dsDNA Assay Kit, underwent bisulfite conversion utilizing the EZ-96 DNA Methylation Kit (Zymo Research). The converted DNA was then analyzed using the Illumina Infinium Human Methylation450K (450K) or the Illumina Infinium MethylationEPIC (EPIC) array, following the manufacturer’s protocols. This process was carried out by the Pennsylvania State College of Medicine Genome Sciences Core facility. To minimize technical variation, DNA samples from ages 9 and 15 were processed concurrently. Samples were randomized to prevent bias. The red and green image pairs were imported into R for analysis. Quality control (QC) of the methylation data was conducted initially using EWAStools. Probes were excluded if their detection values exceeded 0.01 for the 450K array or 0.05 for the EPIC array, or if the number of methylated or unmethylated bead counts was less than four. Probes were also removed based on the ENmix function QCinfo, which was applied with default parameters. Samples were excluded if they had outlier methylation or bisulfite conversion values as identified by the ENmix QC function, or if the predicted sex from the methylation data did not match the recorded sex. Additionally, samples were flagged if sequential samples from the same individual showed genetic discordance between visits. The ENmix preprocessENmix and rcp functions were employed to normalize dye bias, apply background correction, and adjust for probe-type bias [13].

### 2.8. Development of the DNAm GrimAge Epigenetic Clock

The DNAm GrimAge epigenetic clock was created by Ake Lu, Steve Horvath, and their team using data from the Framingham Heart Study. This included DNA methylation data from the HumanMethylation450K BeadChip array, derived from 2356 individuals across 888 pedigrees. Their goal was to construct a mortality risk estimator based on DNA methylation data. Initially, the researchers developed estimators for twelve plasma proteins and smoking pack years using blood methylation data. These estimators, along with chronological age and sex, were then regressed on time-to-death (all-cause mortality) using an elastic net Cox regression model. This model selected seven DNAm-based surrogate plasma protein markers (adrenomedullin [*_adm], beta-2-microglobulin [*_B2M], cystatin C [*_Cystatin_C], growth/differentiation factor 15 [GDF-15; *_GDF_15], leptin [*_leptin], plasminogen activator inhibitor type 1 [PAI-1; *_pai_1], and tissue inhibitor metalloproteinases 1 [TIMP-1; *_TIMP_1]), DNAm pack-years ( *_PACKYRS), chronological age, and sex as covariates. The outcome was converted into years to produce the DNAm GrimAge. AgeAccelGrim represents the residual from regressing observed GrimAge on chronological age. The team also assessed the impact of including imputed blood cell composition in their multivariate Cox regression models. They found that AgeAccelGrim continued to be a strong predictor of lifespan and time-to-coronary heart disease (CHD), even after adjusting for blood cell counts. Except for leptin, where the significance increased with the inclusion of blood cell counts, the adjustments generally reduced significance [13].

### 2.9. Statistical Analysis

Data analysis was conducted using STATA version 18.0. Descriptive statistics, including frequencies (percentages) and means (standard deviations), were reported. Bivariate analysis was performed using the Pearson correlation test. For the multivariable analysis, we applied structural equation modeling (SEM) to examine the associations between race, income to poverty ratio at birth, and youth DNAm Grim Age the outcome 15 years later. The analysis explored the impact of race on biological aging of the child at age 15 via income to poverty ratio at birth. We also controlled for family structure, sex, and ethnicity.

## 3. Results

### 3.1. Descriptive Statistics

A total number of 854 participants entered our analysis. Table 1 shows their descriptive data. 45.32% were Black, 19.32% were Latino, 23.42% were living in married households at baseline, and 50.12% were male.

### 3.2. Bivariate Correlations

Bivariate unadjusted correlations are shown in Table 2. As this table show, DNAm Grim (methylation) was inversely associated with higher SEP and being in a married family. At the same tie, Black race was associated with higher methylation in youth. Male sex was also associated with higher DNAm Grim (methylation).

### 3.3. Multivariable Model, Overall

**Figure 1.**
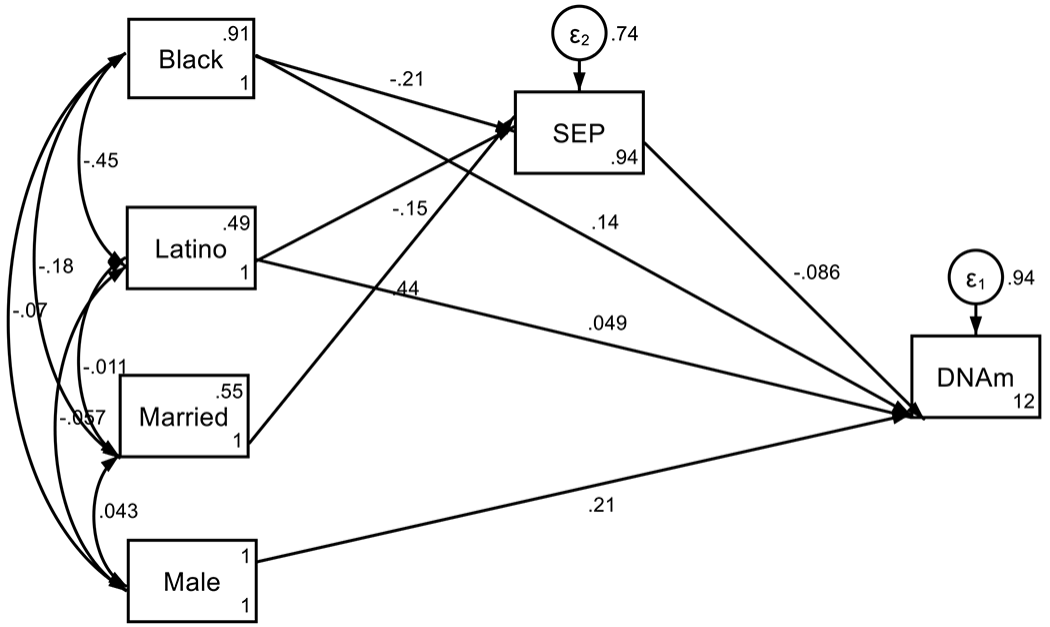
Summary of SEM Overall

Our findings indicate that race was associated with lower SEP at birth as well as faster epigenetic aging. Specifically, income to poverty ratio at birth partially mediated the effects of race on accelerated aging by age 15 (Figure 1, Table 3).

### 3.4. Multivariable Model, Sex-Specific

**Figure 2.**
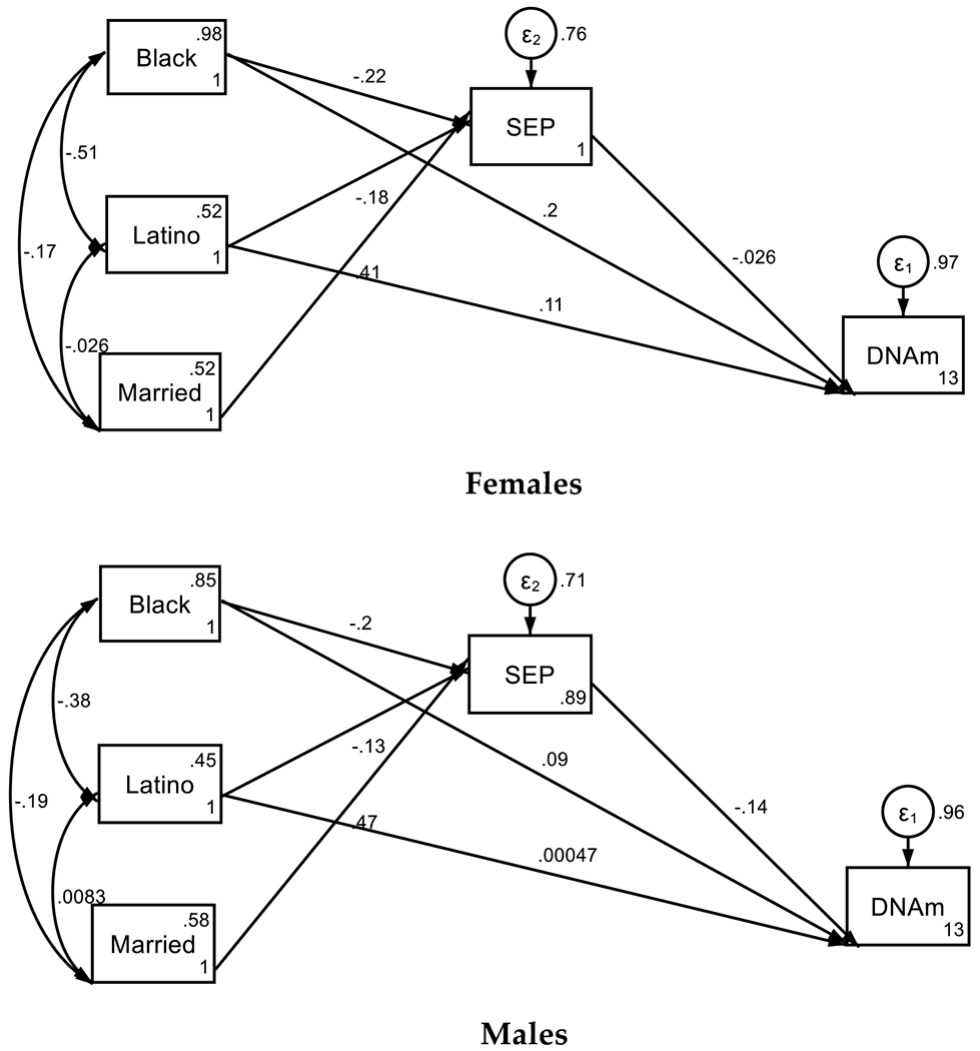
Summary of SEM by Sex

Our findings indicate that SEP at birth was a predictor of faster epigenetic aging of male not female participants in the sample. Specifically, income to poverty ratio at birth partially mediated the effects of race on accelerated aging by age 15 for males but not females (Figure 2, Table 4).

## 4. Discussion

The primary aim of this study was to investigate the associations between race, income to poverty ratio at birth (as our SEP indicator), and epigenetic aging at 15 years of age, using data from the Fragile Families and Child Wellbeing Study (FFCWS). Specifically, we aimed to determine whether SEP (poverty at birth) partially mediates the influence of race on epigenetic aging, as measured by GrimAge, a second-generation epigenetic clock. We also explored sex differences in these associations.

Our findings reveal that being Black is significantly associated with accelerated epigenetic aging by age 15. The study shows that low SEP (higher poverty) experienced at birth partially mediates the effect of race on DNA methylation as measured by GrimAge. This suggests that income to poverty ratio at birth has a lasting impact on biological aging into adolescence among Black individuals. In other words, low SEP is a significant factor in the faster aging observed in youth from Black families. However, the effects of SEP on biological aging were observed in male but not female youth.

Several mechanisms can explain the effects of race on aging. Arline Geronimus, a public health researcher, introduced the “weathering hypothesis” to explain why Black individuals often exhibit signs of accelerated aging compared to their White counterparts [14,15]. This hypothesis posits that the cumulative impact of social, economic, and environmental stressors—rooted in systemic racism—leads to premature biological aging in Black individuals. Geronimus’ theory suggests that chronic exposure to adversity and discrimination experienced by Black communities results in “weathering,” a term she uses to describe the wear and tear on the body due to prolonged stress [16,17]. This concept draws an analogy to how environmental conditions can erode physical structures over time. In the context of human health, the persistent stress and strain from living in a racially stratified society can lead to earlier onset of chronic diseases, higher morbidity, and mortality rates. The weathering hypothesis is supported by substantial evidence showing that Black individuals often have higher levels of stress-related biomarkers, such as cortisol, and exhibit greater physiological dysregulation compared to Whites. This chronic stress leads to increased allostatic load, a measure of the cumulative biological burden exacted by chronic stress and life challenges. Geronimus’ research has shown that these stressors not only affect mental health but also manifest physically, contributing to disparities in birth outcomes, cardiovascular health, and overall mortality [14–22]. The accelerated aging observed in Black individuals is not merely a result of genetics but is significantly influenced by the socio-economic and environmental context shaped by historical and contemporary racism. Her work underscores the importance of addressing the root causes of racial disparities in health by tackling the systemic inequities that perpetuate stress and disadvantage in Black communities. Interventions aimed at reducing these disparities must consider the broader socio-political landscape that influences health outcomes, emphasizing the need for policies that promote social justice and equity.

Race is, in part, a proxy for exposure to racism, and Black individuals are subjected to multi-level discrimination and racism. Due to segregation, Black individuals have lower access to resources and buffers [23–28]. SEP indicators are less protective for Black families than for White families because of systemic racism. Chronic stress, often associated with lower SEP, influences the rate of aging. Financial instability, job insecurity, and social disadvantages increase stress, leading to dysregulation of the hypothalamic-pituitary-adrenal (HPA) axis [29], elevated cortisol levels, and increased allostatic load. Lower SEP affects dietary quality and nutrition. Families with limited access to healthy food options face poor nutrition, which can adversely affect metabolic processes and accelerate aging at the cellular level. Lower SEP is linked with higher exposure to environmental toxins, such as pollutants and lead. Impoverished neighborhoods often have closer proximity to industrial areas and substandard housing, increasing the risk of toxin exposure and inducing epigenetic changes that contribute to accelerated aging. Individuals from lower SEP backgrounds typically have reduced access to healthcare services, resulting in untreated health conditions and delayed diagnoses, which can exacerbate health issues and accelerate aging [30–33].

In our study, we do not view race as a biological factor but rather as a social construct [20, 34–36]. Racial differences observed in health and aging metrics reflect the impact of historical, economic, societal, and environmental influences rather than innate biological distinctions [37–40]. These differences are the result of centuries of systemic inequalities, including discriminatory practices, socioeconomic disparities, and unequal access to resources and opportunities [41–43]. The lived experiences of marginalized racial groups, shaped by persistent exposure to racism and segregation, contribute to the health disparities seen today [44, 45]. By understanding race as a social factor, we aim to highlight the profound effects of structural inequalities and advocate for systemic changes to address these disparities [39, 46–51].

We observed SEP effects on DNA methylation of male not female youth. The effects of SEP on health differs for males and females [52–54]. Sexualized racism and gendered racism highlight the complex interplay between race, gender, and sexuality, illustrating that men and women experience racism in distinctly different ways [55–57]. Sexualized racism refers to the unique discrimination faced by individuals based on both their race and perceived sexual characteristics or stereotypes. For example, women of color often endure hypersexualization and exoticization, leading to objectification and dehumanization in ways that White women do not experience. Conversely, gendered racism emphasizes how racial discrimination manifests differently for men and women. Black men, for instance, may encounter heightened scrutiny and criminalization, often being stereotyped as inherently violent or dangerous. On the other hand, Black women might face stereotypes of being overly aggressive or angry, while also navigating the intersection of sexism and racism in professional and social settings. These intersecting forms of discrimination underscore the necessity of considering both race and gender in understanding the full scope of racism and its varied impacts on different groups [58].

### 4.1. Cautionary Note

In this study, we conceptualized race as a social rather than a biological factor. Although our outcome is a biological measure, our investigation focused on how economic adversities associated with being Black in the U.S. impact biological aging. Specifically, we examined how socioeconomic injustices “get under the skin” and affect cellular aging. Therefore, it would be a misattribution or misunderstanding to interpret race as a biological factor in this context. Our findings underscore the importance of recognizing the social determinants of health and how systemic inequalities contribute to biological outcomes. Interpreting race biologically overlooks the critical role of social environment and economic conditions in shaping health disparities. It is essential to frame our results within the broader context of structural racism and its pervasive effects on health, rather than attributing differences to inherent biological variations among racial groups.

### 4.2. Implications

The findings of this study have significant policy and public health implications. Addressing socioeconomic inequalities at birth is crucial to mitigating the long-term biological impacts of racism on Black families. Policymakers should prioritize public, social, and economic interventions that reduce socioeconomic disparities and improve living conditions for disadvantaged families. Increasing access to quality education, healthcare, and nutritious food, along with reducing exposure to environmental toxins, could help slow down the pace of aging in marginalized communities. Poverty elimination is a major element of addressing racial disparities in accelerated aging. Increasing minimum wage and earned income tax benefits are among the vehicles by which poverty can be reduced in Black families.

### 4.3. Limitations

Several limitations should be considered when interpreting these findings. The study relies on observational data with considerable missing data, which cannot establish causality. Our measure of epigenetic aging was limited to GrimAge, which may require further validation in diverse populations. While the study included income to poverty ratio as the major SEP indicator, several unmeasured confounders could have influenced the observed associations. Long-term follow-up studies have high attrition that may cause bias. Wealth was not included in our analysis, and the poverty variable was only borrowed from the baseline, despite poverty being subject to change. The sample did not include all racial/ethnic groups and is not fully representative of the general U.S. population, limiting the generalizability of the findings. All study variables were measured at the family and individual level, with no neighborhood, community, school, or policy variables used in our analysis.

## 5. Conclusion

This study highlights the long-term impact of early-life SEP on biological aging, with significant implications for health disparities across racial and socioeconomic lines. The association between poverty status at birth and accelerated epigenetic aging underscores the need for comprehensive policies aimed at reducing socioeconomic and racial health inequities from early childhood. Future research should continue to explore the underlying mechanisms and identify effective interventions to mitigate the long-term effects of socioeconomic disadvantages on biological aging. Addressing economic disparities at birth is essential for promoting health equity and improving the overall well-being of disadvantaged populations decades later.

## Figures and Tables

**Table 1.** Shows descriptive data (n = 854).

	n	%
Race (Black)		
No	467	54.68
Yes	387	45.32
Ethnicity (Latino)		
No	689	80.68
Yes	165	19.32
Married Household at Baseline		
No	654	76.58
Yes	200	23.42
Sex (Male)		
No	426	49.88
Yes	428	50.12

**Table 2.** Bivariate Correlations

	1	2	3	4	5	6

1 DNAm Grim (k6mk_pcgrim) at Age 15	1.00					
2 Income to Poverty Ratio (High SEP) at Birth	−0.12*	1.00				
3 Black Race	0.12*	−0.22*	1.00			
4 Latino Ethnicity	−0.02	−0.06*	−0.45*	1.00		
5 Married Household at Birth	−0.09*	0.47*	−0.18*	−0.01	1.00	
6 Male Sex	0.20	0.00	−0.07*	−0.06*	0.04	1.00

DNAm: DNA Methylation, SEP: Socioeconomic Position,

* p < 0.05, Pearson Correlation Test

**Table 3.** Summary of SEM in the Pooled Sample

		OIM			
	Standardized Coefficient	std. err.	[95% conf. interval]		P>z
DNAm Grim (k6mk_pcgrim) at Age 15					
Household Income to Poverty Ratio (SEP) at Birth	−0.09	0.03	−0.15	−0.02	0.013
Black	0.14	0.04	0.06	0.21	< 0.001
Latino	0.05	0.04	−0.03	0.12	0.197
Immune Cells %	0.00	962.00	−1885.49	1885.49	1.000
Male	0.21	0.03	0.14	0.27	< 0.001
Intercept	12.29	0.32	11.66	12.92	< 0.001
Household Income to Poverty Ratio (SEP)					
Black	−0.21	0.03	−0.27	−0.14	< 0.001
Latino	−0.15	0.03	−0.21	−0.09	< 0.001
Married Household at Birth	0.44	0.03	0.38	0.49	< 0.001
Intercept	0.94	0.06	0.82	1.05	< 0.001

**Table 4.** Summary of SEM by Sex

	Standardized Coefficient	std. err.	95%	CI	p
**Females**					
Structural					
DNAm Grim (k6mk_pcgrim)					
Household Income to Poverty Ratio (SEP) at Birth	−0.03	0.05	−0.12	0.07	0.604
Black	0.20	0.06	0.09	0.31	< 0.001
Latino	0.11	0.06	0.00	0.22	0.047
Immune Cells %	12.93	0.47	12.00	13.85	< 0.001
Household Income to Poverty Ratio (SEP) at Birth					
Black	−0.22	0.05	−0.32	−0.13	< 0.001
Latino	−0.18	0.05	−0.27	−0.08	< 0.001
Immune Cells %	0.41	0.04	0.33	0.48	< 0.001
Intercept	1.00	0.09	0.82	1.18	< 0.001
**Males**					
Structural					
DNAm Grim (k6mk_pcgrim)					
Household Income to Poverty Ratio (SEP) at Birth	−0.14	0.05	−0.24	−0.05	0.003
Black	0.09	0.05	−0.02	0.19	0.093
Latino	0.00	0.05	−0.10	0.10	0.993
Immune Cells %	12.57	0.44	11.71	13.42	< 0.001
					
Household Income to Poverty Ratio (SEP) at Birth					
Black	−0.20	0.04	−0.29	−0.11	< 0.001
Latino	−0.13	0.04	−0.22	−0.05	0.002
Immune Cells %	0.47	0.04	0.39	0.54	< 0.001
Intercept	0.89	0.08	0.74	1.05	< 0.001

## Data Availability

FFCWS data are available to public at Office of Population Research data repository available at https://oprdata.princeton.edu.
